# MSH3-Deficiency Initiates EMAST without Oncogenic Transformation of Human Colon Epithelial Cells

**DOI:** 10.1371/journal.pone.0050541

**Published:** 2012-11-27

**Authors:** Christoph Campregher, Gerald Schmid, Franziska Ferk, Siegfried Knasmüller, Vineeta Khare, Benedikt Kortüm, Kyle Dammann, Michaela Lang, Theresa Scharl, Andreas Spittler, Andres I. Roig, Jerry W. Shay, Christopher Gerner, Christoph Gasche

**Affiliations:** 1 Christian Doppler Laboratory for Molecular Cancer Chemoprevention, Division of Gastroenterology and Hepatology, Department of Medicine 3, Medical University of Vienna, Vienna, Austria; 2 Institute of Cancer Research, Department of Medicine I, Medical University of Vienna, Vienna, Austria; 3 ACIB GmbH, c/o Department of Biotechnology, University of Natural Resources and Life Sciences, Vienna, Austria; 4 Department of Statistics and Probability Theory, University of Technology, Vienna, Austria; 5 Department of Surgery, Research Laboratories & Core Facility Flow Cytometry, Medical University of Vienna, Vienna, Austria; 6 Department of Cell Biology, Division of Digestive and Liver Diseases, University of Texas Southwestern Medical Center, Dallas, Texas, United States of America; 7 Department of Medicine I, Comprehensive Cancer Center, Medical University of Vienna, Vienna, Austria; Baylor University Medical Center, United States of America

## Abstract

**Background/Aim:**

Elevated microsatellite instability at selected tetranucleotide repeats (EMAST) is a genetic signature in certain cases of sporadic colorectal cancer and has been linked to MSH3-deficiency. It is currently controversial whether EMAST is associated with oncogenic properties in humans, specifically as cancer development in Msh3-deficient mice is not enhanced. However, a mutator phenotype is different between species as the genetic positions of repetitive sequences are not conserved. Here we studied the molecular effects of human MSH3-deficiency.

**Methods:**

HCT116 and HCT116+chr3 (both MSH3-deficient) and primary human colon epithelial cells (HCEC, MSH3-wildtype) were stably transfected with an EGFP-based reporter plasmid for the detection of frameshift mutations within an [AAAG]17 repeat. MSH3 was silenced by shRNA and changes in protein expression were analyzed by shotgun proteomics. Colony forming assay was used to determine oncogenic transformation and double strand breaks (DSBs) were assessed by Comet assay.

**Results:**

Despite differential MLH1 expression, both HCT116 and HCT116+chr3 cells displayed comparable high mutation rates (about 4×10^−4^) at [AAAG]17 repeats. Silencing of MSH3 in HCECs leads to a remarkable increased frameshift mutations in [AAAG]17 repeats whereas [CA]13 repeats were less affected. Upon MSH3-silencing, significant changes in the expression of 202 proteins were detected. Pathway analysis revealed overexpression of proteins involved in double strand break repair (MRE11 and RAD50), apoptosis, L1 recycling, and repression of proteins involved in metabolism, tRNA aminoacylation, and gene expression. MSH3-silencing did not induce oncogenic transformation and DSBs increased 2-fold.

**Conclusions:**

MSH3-deficiency in human colon epithelial cells results in EMAST, formation of DSBs and significant changes of the proteome but lacks oncogenic transformation. Thus, MSH3-deficiency alone is unlikely to drive human colon carcinogenesis.

## Introduction

Microsatellite instability (MSI) is a hallmark of tumors in patients with Lynch syndrome and can be detected in about 15% of all colorectal cancers [1]. Frameshift mutations within microsatellite sequences are caused by DNA polymerase slippage followed by a dysfunction of the mismatch repair (MMR) system [2], [3]. A certain phenotype of MSI named EMAST (elevated microsatellite alterations at selected tetranucleotide repeats) has been observed in non-small cell lung [4], [5], skin [6], ovarian [7], urinary tract [8], prostate [9], [10], bladder [6], [11], and recently colorectal cancer (CRC) [12]–[16]. However, the molecular basis for EMAST is incompletely understood. There is evidence for a rare association of EMAST with mutations in MLH1 and MSH2 in endometrial cancer [17]. EMAST is commonly found in sporadic CRC and an overlapping mechanism may exist between MSI-low, EMAST, and loss of heterozygosity [12]. In CRC, MSH3-deficiency is associated with EMAST and MSI at dinucleotide repeats [12]. MSH3 itself is a known target of frameshift mutations at its [A]8 repeat in exon 7, which results in loss of MSH3 expression [18], [19]. Among tetranucleotide repeats the [AAAG]n motif represents the majority in the human genome [20]. Such repeats are prone to frameshift mutagenesis, therefore highly polymorphic and used as biomarkers for certain cancers [21]–[23].

Cancer cells often exhibit a mutator phenotype as a result of mutations in genes that maintain genomic integrity, thereby driving the genetic evolution of cancer cells [24]. So far, a direct link between EMAST as a mutator phenotype has not been established [25]. In mice, Msh3 deficiency alone did not cause cancer predisposition, but a simultaneous loss of Msh3 and Msh6 accelerated intestinal tumorigenesis while lymphomagenesis was not affected [26]. The incidence of lymphomas in Msh6-deficient mice was as high as in Msh2-deficient mice while in Msh6-deficient mice the development of intestinal tumors was rare compared to Msh2-deficient mice [26]. Msh3-wildtype as well as Msh3-deficient mice developed tumors with similar incidence at 2-years of age [27]. Msh3-deficient mice developed a few gastrointestinal tumors (similar to Msh2-, Mlh1-, and Msh6-deficient mice), but due to the small number of tumors it was not possible to conclude that the absence of Msh3 represents a separate mutator phenotype [27]. MSH3 mRNA was not detectable in hematologic progenitor cells of patients with lymphocytic and myelogenous leukemia suggesting that inactivation of the MSH3 gene may be involved in the development of hematologic malignancies [28]. The association of EMAST with immune cell infiltration in rectal cancer suggests a role of inflammation in the development of EMAST [16], [29]. It is currently controversial whether EMAST or loss of MSH3 alone is associated with oncogenic transformation in human colon epithelial cells. A study by Plaschke et al. suggested that MSH3 abrogation may be a predictor of metastatic disease or even favors tumor cell spreading in MLH1-deficient CRC [18]. In contrast, a recent study by Laghi et al. revealed that MLH1-deficient CRCs not expressing MSH3 have more severe MSI, but a lower rate of nodal involvement, and a better postsurgical outcome [30]. Furthermore, CRC-patients exhibiting MSI-L and/or EMAST had shorter times of recurrence-free survival than patients with MSI-H and hypoxia is suggested to be a mechanistic link between MSI-M (moderate levels of microsatellite instability) and recurrent metastasis [31].

MSH3 interacts with MSH2 to form the mispair-binding complex MutSβ [32]. MSH3 contains an N-terminal sequence motif characteristic of proteins which bind to proliferating cell nuclear antigen (PCNA), and this interaction may facilitate early steps in DNA mismatch repair [33]. MSH3 also directly and indirectly interacts with breast cancer susceptibility gene product (BRCA1) and BRCA1-associated RING domain protein 1 (BARD1) which may partially provide an explanation for the background of gynecological and CRC in both Lynch syndrome and BRCA1 individuals [34]. Interaction domains also exist between MSH3 and human exonuclease I (hExoI), a family member of conserved 5′ → 3′ exonucleases. Such interaction suggests an involvement of hExoI as a downstream effector in MMR and/or DNA recombination [35]. MMR-deficiency is not only limited to mutation or transcriptional silencing of MMR-genes, but can also be the result of an imbalance in the relative expression levels of MSH3 or MSH6 [36]. Loss of MSH3 was associated with increased chemotherapeutic activity of platinum drugs [37]. MutSβ is also involved in the process of CAG repeat expansion [38] as well as the repair of isolated short CTG/CAG DNA slip-outs [39].

Previously we developed an EGFP-based assay for the quantitation of frameshift mutations within mono-, and dinucleotide repeats in HCT116 and HCT116+chr3 colon epithelial cells [40]. Herein we extended this model to study frameshift mutations within an [AAAG]17 tetranucleotide repeat in HCT116, HCT116+chr3 and in primary colonic epithelial cells (HCEC-1CT). Furthermore, we investigated the effect of MSH3-silencing on oncogenic transformation and on the proteome.

## Results

### Establishment and Characterization of Reporter Cell Lines

**Figure 1:**
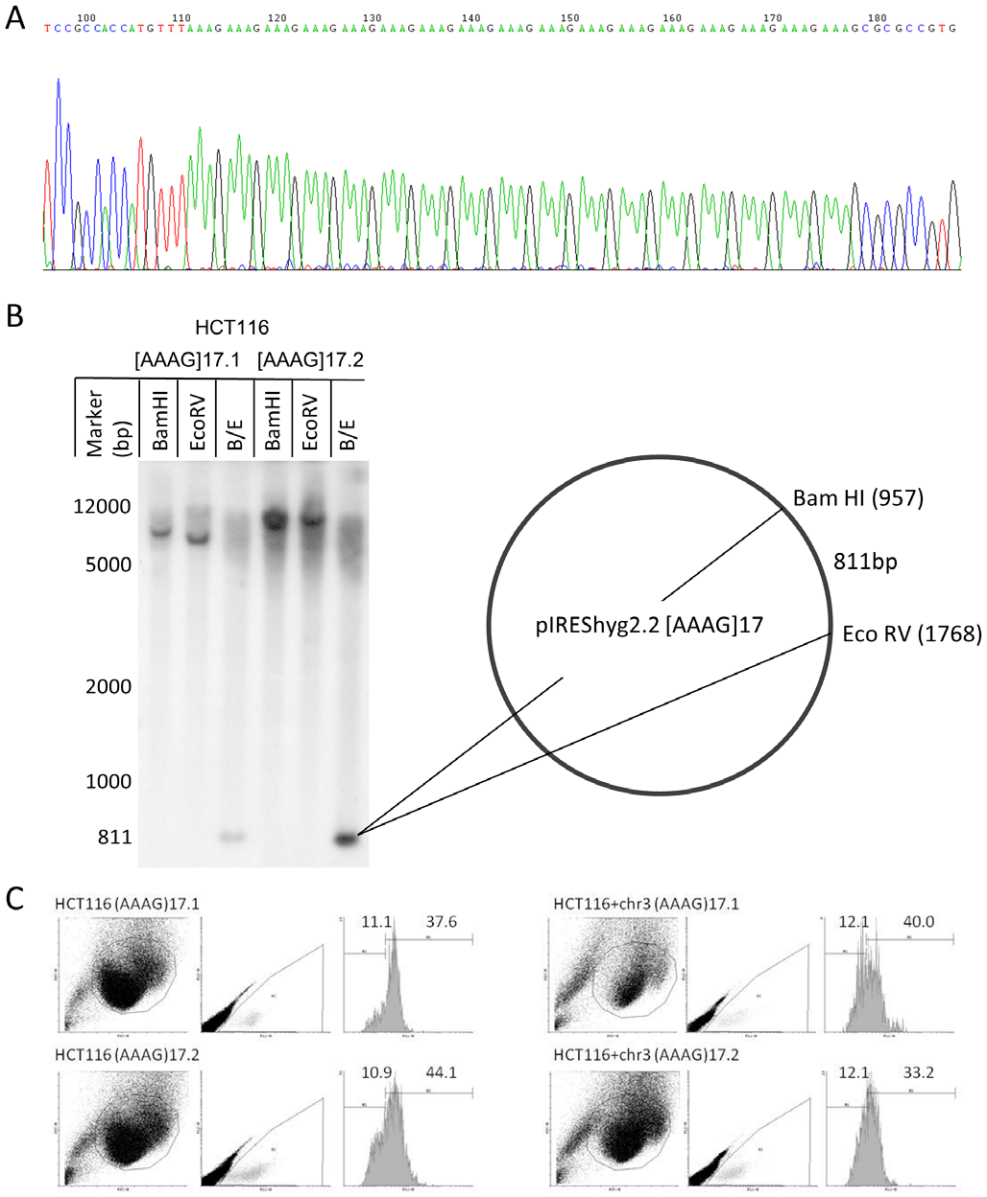
Characterization of frame-shift reporter-plasmid and -cell lines. (A) Sequence analysis of pIREShyg2-EGFP-[AAAG]17 plasmid. Genomic DNA was isolated, the EGFP region containing the [AAAG]17-repeat was amplified by PCR and sequenced. (B) Verification of plasmid insertion number by Southern blot analysis. 20µg of total DNA was digested with BamHI, EcoRV, or both (B/E), resolved on a 0.8% agarose gel, and transferred onto a nylon membrane. Complementary EGFP-DNA was labeled with [P-32]-dCTP, hybridized, and the blots were analyzed by autoradiography. The 811bp fragment (harboring the coding region for EGFP with the [AAAG]17 microsatellite] is generated by restriction with BamHI (position 957) and EcoRV (position 1768). (C) Flow cytometric analysis of unsorted HCT116-[AAAG]17 and HCT116+chr3-[AAAG]17 cell clones showing similar accumulation of EGFP-positive (mutated) cells and fluorescence intensities (the geometric mean of the FL1 intensity was expressed as mean +SD for the M1 and the M2 fractions).

**Figure 2:**
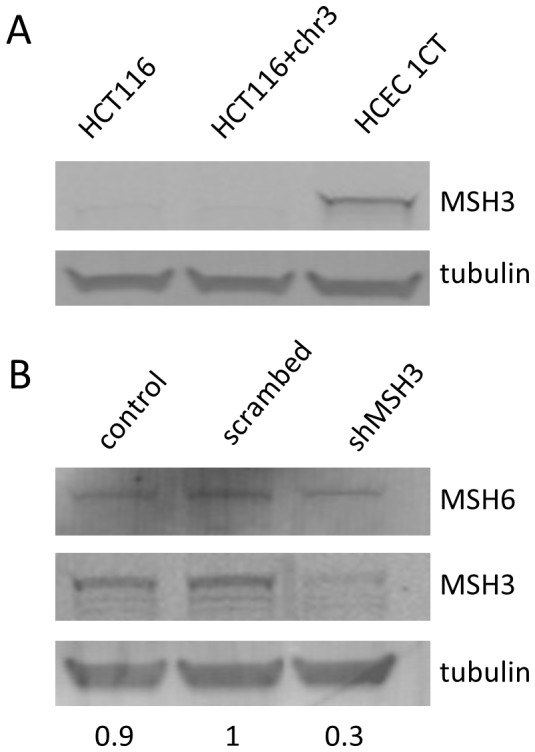
MMR-protein expression and MSH3-silencing in colon epithelial cells. (A) Western blot analysis of HCT116, HCT116+chr3 and HCEC-1CT cells. (B) Silencing of MMR-protein MSH3 by gene-specific shRNA in HCEC-1CT-[AAAG]17 cells resulted in 70% repression of MSH3. MSH6 was not affected.

The plasmid pIREShyg2-EGFP allows the expression of EGFP under the control of a CMV promoter. An oligo with the repeated sequence [AAAG]17 was inserted after the start codon of the EGFP, thereby shifting it out of frame (Figure 1A). The previously established reporter plasmids pIREShyg2-EGFP-[CA]13 and pIREShyg2-EGFP-[N]26 (a random non-repeat sequence) [40] served as controls. Deletions or insertions within the repetitive sequence may restore the proper reading frame of EGFP. MLH1-proficient (HCT116+chr3) and MLH1-deficient cells (HCT116) were transfected with pIREShyg2-EGFP-[CA]13 and pIREShyg2-EGFP-[AAAG]17 as previously described [40]. Stable single cell clones were established and characterized by sequencing, Southern blot (Figure 1B) and flow cytometry (Figure 1C). A similar approach was carried out using primary colonic epithelial cells (HCEC-1CT) [41] resulting in HCEC-1CT-[AAAG]17 and HCEC-1CT-[CA]13. As HCECs need a certain cell density and cell to cell contact for expansion, we were unable to perform single cell cloning; therefore, we used stable mixed populations. HCT116 and HCT116+chr3 lack MSH3 which is crucial for the repair of frameshift-mutations in tetranucleotide repeats [12] while HCEC express MSH3 (Figure 2A).

### MSH3 Maintains Stability of [AAAG]17 Repeats

Complementation of MLH1-deficiency by chromosome 3 transfer stabilizes [A]n and [CA]n repeats but fails to stabilize [AAAG]n repeats [12]. Loss of MSH3 causes EMAST and low level MSI (MSI-L) in dinucleotide repeat sequences [12], [15]. We utilized the model described above to compare the stability of [AAAG]17 repeats in HCT116 and HCT116+chr3, as well as in HCEC.

In contrast to [CA]13 (data from [40]), the mutation rate of the [AAAG]17 repeat was high in both HCT116 and HCT116+chr3 cells (Table 1). Sequencing of the [AAAG]17 repeat in single cell clones of the EGFP-negative M0 fraction revealed a heterogeneous population of wildtype and 1-unit deletions in clones HCT116-[AAAG]17.1, HCT116+chr3-[AAAG]17.1 and HCT116+chr3-[AAAG]17.2 and exclusive 1-unit deletions in clone HCT116-[AAAG]17.2 (all being non-fluorescent; Table 2) reflecting the speed of mutations during clonal expansion. In the M1 fraction one intermediate mutant cell (0/+1) was detected in clone HCT116+chr3-[AAAG]17.1 [42]. Several cells from the EGFP-positive M1 and M2 populations harbored mutations which would result in non-fluorescent cells. In the M2 population of HCT116-[AAAG]17.1 we also discovered a sub-clone with a 2 bp (AA) deletion (Table 2), indicating that not only gain or losses from full repeat units may occur but also disruptions of single units, at least in MLH1-deficient HCT116 cells. Overall, we observed clonal variations rather than differences between HCT116 and HCT116+chr3 cells indicating that loss of MSH3 is likely to be the cause (Table 2).

**Table 1:** Mutation rates within tetra- and dinucleotide repeats.

clone	HCT116		HCT116+chr3		p-value*
	ML	MM	ML	MM	
[AAAG]17.1**	5.2±0.7 (×10^−4^)	5.4±1.0 (×10^−4^)	3.1±0.6 (×10^−4^)	6.7±2.0 (×10^−4^)	n.s.
[AAAG]17.2**	2.4±0.4 (×10^−4^)	2.3±0.7 (×10^−4^)	3.1±0.5 (×10^−4^)	4.7±1.3 (×10^−4^)	n.s.
[CA]13.1	2.0±0.3 (×10^−4^)	1.9±0.5 (×10^−4^)	9.0±4.4 (×10^−6^)	8.9±5.5 (×10^−6^)	<0.001

Data are mean ± SEM.

Mutation rates are expressed as mutations per microsatellite per generation [42], [73].

ML. maximum likelihood method.

MM. method of the mean.

* Between mutation rates (MM) of MMR-deficient (HCT116) and MMR-corrected (HCT116+chr3).

** Mutation rates may be underrated as clones are a mix of [AAAG]17 and 1-unit deletion mutants (as illustrated in the M0 fraction in Table 2).

n.s. not significant.

**Table 2:** Mutation spectrum within tetranucleotide repeats.

cells	clone	M0		M1							M2			
		−1	0	−2	−1/−2	−1	0/−1	0	0/1	1	−2	−1	0	1
HCT116	[AAAG]17.1	14	12	8		5		2		1	21			3
	[AAAG]17.2	10		9		3					6	5*		
HCT116+chr3	[AAAG]17.1	3	13	1		1		4	1	5	7	1	1	6
	[AAAG]17.2	7	3	2		2				10	6	1		11

−2, −1, 1. Change in the number of [AAAG]-units (e.g. −2 =  loss of 8 bp or [AAAG]2).

−1, 0: Wildtype or frameshift mutant cells without expected EGFP fluorescence.

−2, 1: In-frame mutant cells with expected EGFP fluorescence.

−1/−2, 0/1: Heteroduplex mutant cells with expected partial (dim) EGFP fluorescence.

* including one mutant with a 2 bp (AA) deletion.

**Figure 3:**
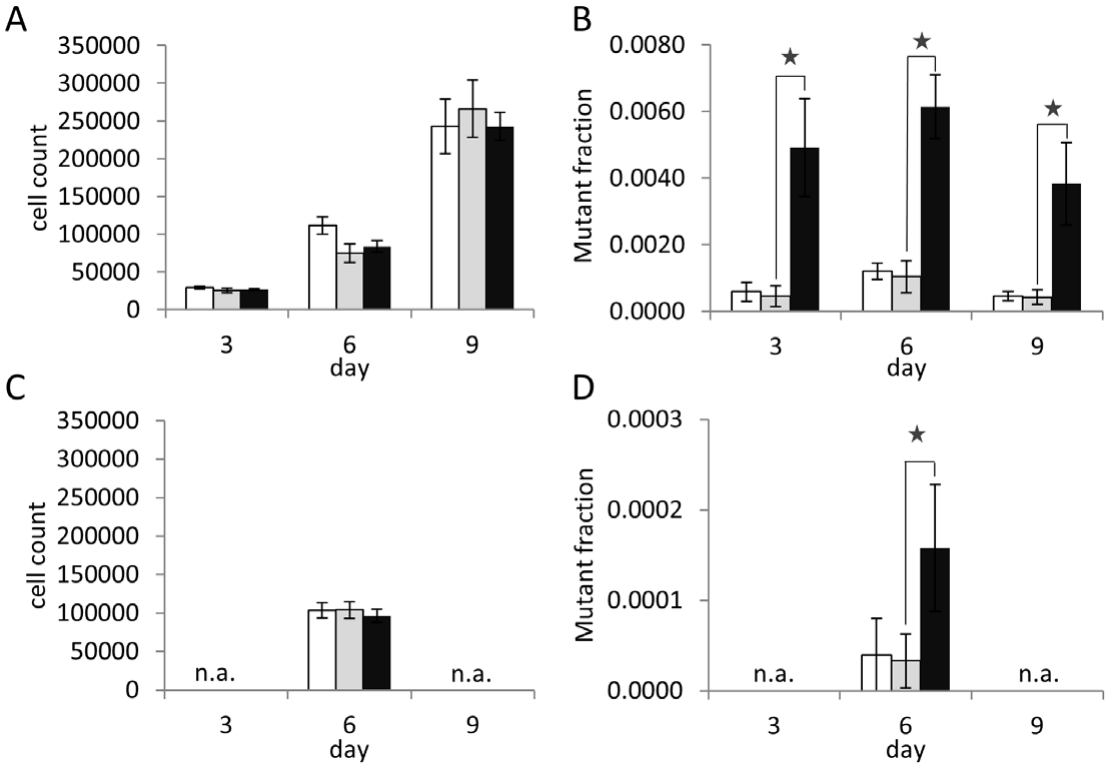
Analysis of frame-shift mutations in [AAAG]17- and [CA]13-repeats in MSH3-silenced primary colon epithelial cells. 2×10^4^ EGFP-negative/RFP-positive cells were sorted into 24-well plates and mutant fractions were analyzed after 3, 6 and 9 days. At day 4 cells were expanded to 6-well plates to sustain exponential growth (A) Cell count of HCEC-1CT-[AAAG]17-control, HCEC-1CT-[AAAG]17-scrambled and HCEC-1CT-[AAAG]17-shMSH3 cells at day 3, 6 and 9 revealed exponential growth. (B) Analysis of EGFP-positive mutant fractions at day 3, 6 and 9 showed a 6- to 10-fold elevated mutant fraction in HCEC-1CT-[AAAG]17-shMSH3 cells compared to HCEC-1CT-[AAAG]17-scrambled. No difference was observed between HCEC-1CT-[AAAG]17-scrambled and HCEC-1CT-[AAAG]17-control cells. (C) Cell count of HCEC-1CT-[CA]13-control, HCEC-1CT-[CA]13-scrambled and HCEC-1CT-[CA]13-shMSH3 cells at day 6. (D) Analysis of EGFP-positive mutant fractions at day 6 showed a 3-fold increase in HCEC-1CT-[CA]13-shMSH3 cells compared to HCEC-1CT-[CA]13-scrambled. No difference was observed between HCEC-1CT-[CA]13-scrambled and HCEC-1CT-[CA]13-control cells. Mean of triplicate cultures for each clone, bars, SE. Asterisks indicate statistical significance of p<0.05. n.a. = not analyzed. White bars = control, grey bars = scrambled, black bars = shMSH3.

To better understand the role of MSH3 in EMAST we studied the effect of MSH3 silencing in [AAAG]17-transfected HCEC (Figure 2A). RNA silencing in HCEC resulted in repression of MSH3 by 70% (Figure 2B). No differences in MSH3 expression were observed between HCEC transfected with control and scrambled shRNA-plasmids. In addition, MSH6 expression was not affected by MSH3 silencing. EGFP-negative cells were sorted and cultured for up to 9 days. The proliferation of HCEC-1CT-[AAAG]17-shMSH3 was comparable to HCEC-1CT-[AAAG]17-scrambled or HCEC 1CT-[AAAG]17-control cells (Figure 3A). At day 3 the EGFP-positive mutant fraction in HCEC 1CT-[AAAG]17-shMSH3 was about 10-fold higher than in HCEC 1CT-[AAAG]17-scrambled cells (Figure 3B). This mutant fraction did not continually increase after day 3 suggesting that simultaneous forward and reverse mutations occur in culture after reaching a fast plateau.

In order to check whether lack of MSH6, a member of the MutSα complex, also influences the stability of [AAAG]17 we compared mutant fractions of [AAAG]17 or [CA]13 repeats with a non-repetitive sequence [N]26 [40] in MSH6-deficient DLD-1 cells. Mutations at the [AAAG]17 repeat were significantly higher (14-fold, p<0.05) while mutations at the [CA]13 repeat did not differ from mutations within the [N]26 sequence (Figure S1) suggesting a possible role of MSH6 in the repair of [AAAG]17. As expected, HCT116 cells revealed higher levels of instability at both microsatellites. However, when compared to DLD-1 cells, the level of [AAAG]17 instability was only about 5-fold higher indicating that MSH6 deficiency alone causes rather low levels of MSI at tetranucleotide repeats.

As previous studies indicated that EMAST is also associated with low level instability of dinucleotide repeats [12], we also transfected a [CA]13 dinucleotide repeat into HCEC. Silencing of MSH3 did not have an effect on proliferation (Figure 3C). However, the mutant fraction in HCEC 1CT-[CA]13-shMSH3 increased about 4-fold at day 6 (Figure 3D). Taken together, these results indicate that loss of MSH3 results in increased instability at [AAAG]17 and to a certain degree at [CA]13 repeats. This suggests that MSH3-deficiency affects tetranucleotide stability to a higher extent than dinucleotide stability.

### MSH3-deficiency does not Trigger Oncogenic Transformation

Inactivation or loss of a single protein may trigger tumor development. This is specifically true for MMR proteins including MLH1, MSH2 and MSH6. Our data and others [12], [15] demonstrate that MSH3-deficiency induces EMAST. However, it is unclear whether loss of MSH3 has oncogenic properties. In fact, exonic tetranucleotide repeats are uncommon in the human genome and only seven are located in coding regions [25]. Here we studied the effect of permanent MSH3 silencing to test whether this alters cellular pathways which may affect tumor development. Proteome analysis was performed by shotgun analysis of nuclear and cytoplasmic fractions from HCEC-1CT, HCEC 1CT-[AAAG]17-scrambled, HCEC-1CT-[AAAG]17-shMSH3, and HCEC-1CT-shMSH3. Data were pooled from HCEC-1CT and HCEC-1CT-[AAAG]17-scrambled (Pool A, analysis set) and from HCEC-1CT-[AAAG]17-shMSH3 and HCEC-1CT-shMSH3 (Pool B, reference set). Proteome analysis resulted in a total of 2215 proteins generated of 24410 peptide IDs containing a false rate of less than 1%.

Stable suppression of MSH3 in primary colon epithelial cells caused an at least 2-fold overexpression of 29 nuclear and 27 cytoplasmic proteins. Plectin-1, a major cytoskeleton cross-linking protein that binds to actin was overexpressed about 7-fold in both fractions. *De novo* expression of 15 nuclear and 6 cytoplasmic proteins was induced by MSH3-silencing. A total of 8 nuclear and 90 cytoplasmic proteins were repressed at least 2-fold and complete loss of 6 nuclear and 15 cytoplasmic proteins was caused by MSH3-silencing. 5 proteins (Peptidyl-prolyl cis-trans isomerase H; Protein BUD31 homolog; Histone H1x; Peptidyl-prolyl cis-trans isomerase NIMA-interacting 4; AP-3 complex subunit beta-1) were completely lost in both the nuclear and the cytoplasmic fraction (for a complete list see also Table S1).

**Figure 4:**
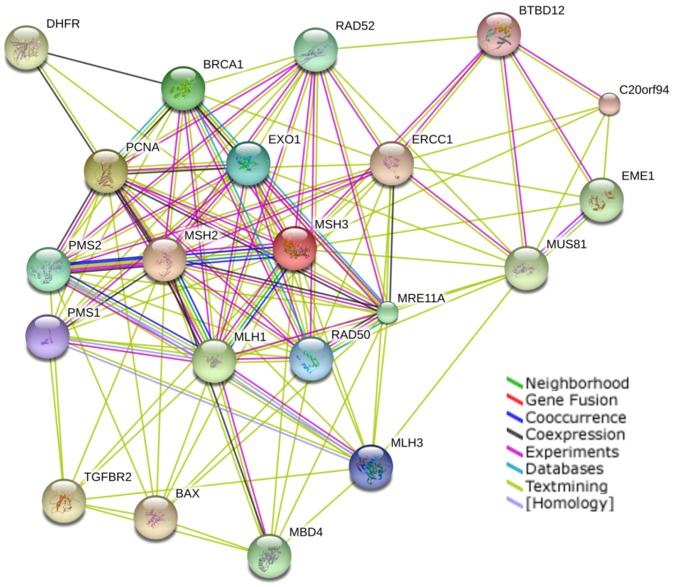
Interaction partners of MSH3. MSH3-Interaction partners were analyzed by string-db-org (http://string-db.org) set to a medium confidence of 0.400 using the active prediction mMethods; Neighborhood, Gene Fusion, Co-occurrence, Co-expression, Experiments, Databases, and Textmining (see legend).

Proteins levels which were changed 2-fold were assessed by reactome pathway analysis [43] (http://www.reactome.org) using the algorithm for “over-representation analysis” resulting in a list of statistically over-represented pathways. The best mapping pathways (with a p-value<10^−4^) of the 78 over- and *de novo*-expressed proteins are the recycling pathway of L1 (p<10^−4^, 5/25 proteins), apoptosis (p<10^−4^, 7/137) and the formation of the RAD50:MRE11 complex (p<10^−4^, 2/2). Repressed and completely lost proteins significantly mapped metabolism- (p<10^−6^, 30/851), tRNA aminoacylation- (p<10^−5^, 7/42), and gene expression pathways (p<10^−6^, 18/413) (Tables S2 and S3). Among the molecular interaction partners of MSH3 (Figure 4, STRING 9.0 web server http://string-db.org/ [44] was used to generate the molecular association network) only the double-strand break repair protein MRE11A (meiotic recombination 11 homolog A), the DNA repair protein RAD50 and the apoptosis regulator BAX (BCL2-associated × protein) were found to be induced. MRE11A and RAD50 were *de novo* induced in the nucleus while BAX was overexpressed in the cytoplasm. None of the tetranucleotide harboring proteins [DUX4 (NM_033178), LOC389328 (XM_374137), LOC284895 (XM_209398), LOC285221 (XM_209521), LOC286039 (XM_209873), LOC284934 (XM_211696), or LOC440175 (XM_498577)] [25] were detectable in HCEC. In summary, our proteome data show that repression of a single MMR protein, MSH3, induces significant changes in the expression of 202 proteins which are involved in fundamental cellular pathways.

### MSH3-silencing does not Induce Oncogenic Transformation of HCECs

**Figure 5:**
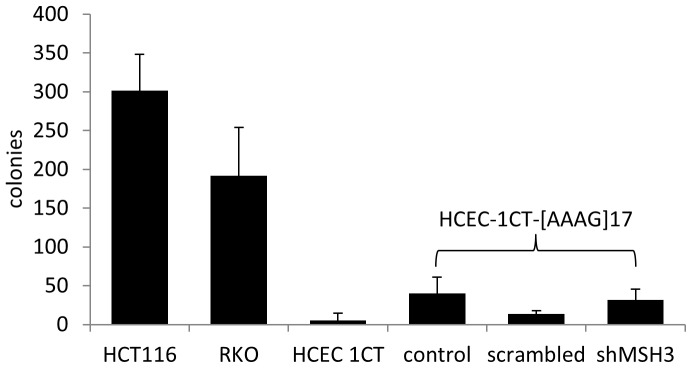
MSH3-silencing does not induce oncogenic transformation in HCECs. Colony forming assay was used to estimate oncogenic transformation in HCECs upon MSH3-silencing. 5×10^3^ cells were seeded in 6-well plates in soft agar and colonies were counted. The positive-controls HCT116 and RKO produced numerous colonies whereas hTERT/Cdk4 transduced HCEC-1CT produced only five colonies similar to HCEC-1CT-[AAAG]17-scrambled. HCEC-1CT-[AAAG]17-control and HCEC-1CT-[AAAG]17-shMSH3 produced 40 and 32 colonies, respectively. Data represent mean ± SD from three experiments.

Loss of single MMR-proteins can lead to cancer development [45]. Data from our shotgun proteomic approach suggest that loss of MSH3 alone does not trigger oncogenic transformation in HCECs. To further support this we used a soft agar assay. HCT116 and RKO cells served as positive controls and revealed formation of 301±47 and 192±62 colonies, respectively. HCEC-1CT produced only five colonies (±9) similar to HCEC-1CT-[AAAG]17-scrambled (13±5). HCEC-1CT-[AAAG]17-control and HCEC-1CT-[AAAG]17-shMSH3 produced 40±21 and 32±14 colonies, respectively (Figure 5). We conclude that silencing of MSH3 alone does not induce oncogenic transformation in HCECs.

### MSH3-silencing Leads to Increased Double Strand Breaks in HCECs

**Figure 6:**
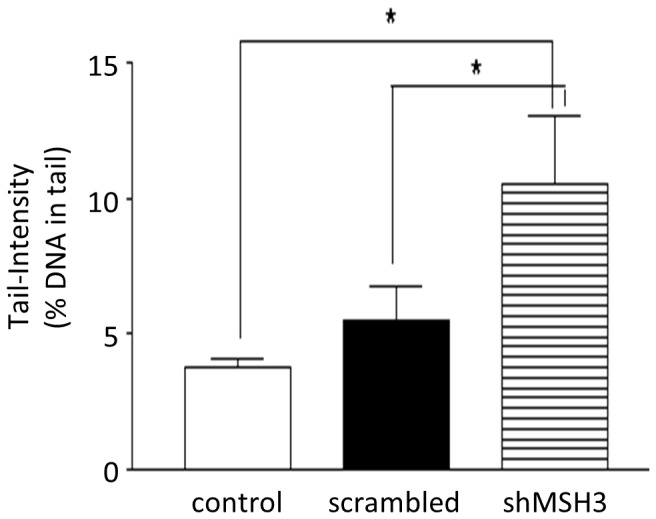
MSH3-silencing leads to increased double strand breaks in HCECs. MSH3 was silenced in HCECs and DSBs were analyzed by SCGE. For each experimental point, three cultures were processed. From each culture three slides were prepared and from each slide 50 cells were analyzed for comet formation. Data represent mean ± SD from one experiment. Stars indicate statistical significance (P<0.05).

MSH3-deficient cancer cells maintain higher levels of phosphorylated histone H2AX and 53BP1 after oxaliplatin treatment in comparison with MSH3-proficient cells, suggesting that MSH3 plays an important role in repairing DNA double strand breaks (DSBs) [37]. Our proteomics data revealed overexpression of RAD50 and MRE11 upon MSH3-silencing indicative for the induction of DSBs. To check for the presence of DSBs we performed a comet assay in HCEC-1CT-[AAAG]17-control, HCEC-1CT-[AAAG]17-scrambled and HCEC-1CT-[AAAG]17-shMSH3. As expected, DSBs significantly increased (2-fold) upon silencing of MSH3 (Figure 6) suggesting a role for MSH3 in the repair of DSBs.

## Discussion

MSI was primarily described in cancer of the proximal colon [46] and subsequently found in tumors from patients with Lynch syndrome at mono- and dinucleotide repeats [47], [48]. Another appearance of MSI, EMAST, which occurs at tetranucleotide repeats was described primarily in non-small cell lung cancer [4]. It is still questionable whether EMAST is an innocent bystander phenomenon or represents a particular mutator pathway. The data of this study support the notion that MSH3 silencing is sufficient to induce EMAST but insufficient to transform human colon epithelial cells *in vitro*. Also there is conflicting data on the effect of EMAST on the prognosis, survival or outcome in patients with cancer. According to our *in vitro* studies MSH3-silencing does not alter the proteome in favor of tumor growth, invasion, or metastasis [49]. The results may partially explain why Msh3-knockout mice exhibit normal life-span and only late onset of tumor development [27], or why germline mutations of MSH3 are not detected in families with Lynch syndrome [50]. Thus, loss of MSH3 alone is unlikely to drive colon carcinogenesis in humans [18].

We are not the first to link MSH3-deficiency and EMAST. In a previous study the EMAST markers MYCL1, D20S82, D20S85, L17835, D8S321, D9S242 and D19S394 where analyzed in MSH3-silenced or MSH3-deficient cells which exhibited EMAST and low levels of dinucleotide mutations [12]. However, these colorectal cancer-derived cell lines were corrected by transfer of complete chromosomes and carried a variety of mutations. Therefore, certain conclusions may have been indirect. Here we utilized primary, non-mutated colon epithelial cells [41] in which MSH3 was selectively silenced. By applying a specific reporter assay [40], [42] as sensitive quantitation of frameshift mutations we observed comparable mutation rates in HCT116 and HCT116+chr3 cells [12]. Sequence analysis of the actual tetranucleotide mutations revealed high heterogeneity (insertions and deletions) and only a single intermediate mutant, which is likely to reflect the hypermutable phenotype in the absence of MSH3. In fact, the mutation rate within the [AAAG]17 repeat is as high as the estimated rate of polymerase slippage, which suggests that loss of MSH3 leads to a complete loss of [AAAG]17 mutation repair [51]. Some instability was also detected at [CA]13 repeats confirming previous data [12]. Interestingly, also MSH6-deficient DLD1 cells displayed some instability at [AAAG]17 but not at [CA]13 repeats. It has been suggested that MSH6-deficiency was only associated with the instability of mononucleotide repeats [52]. Our data suggest that, to a certain extent, MSH6 is also involved in the repair of [AAAG]17 repeats but not [CA]13 repeats. This is in line with a study by Umar et al. which revealed that MSH6 can participate in the repair of replication errors within tetranucleotide repeats [66].

The mutation rate at the [AAAG]17 repeat in HCT116 and HCT116+chr3 cells seems to be somewhat higher than at the [CA]13 repeat (Table 1). The calculated mutation rates for the [AAAG]17 repeat may even underestimate the actual mutation rate as deletion mutations are present at high levels in the M0 fraction (Table 2), a feature that had not been observed with other repeats [40]. In fact, both insertion and deletion mutations are found in culture (Table 2) which likely reflects a steady state of insertions and deletions. These considerations may partially explain the plateauing of the [AAAG]17 mutant fraction already after few generation cycles (Figure 3). The molecular and structural mechanism of MutSβ in the repair of tetranucleotide frameshifts is still unknown. Non-B DNA secondary structures (formed by tetranucleotide repeats) were discussed as target for the MutSβ complex [53].

Proteome analysis upon MSH3-silencing revealed significant changes in the expression pattern of 202 proteins within 6 cellular pathways. MRE11, a member of the Mre11/RAD50/NBS1 (MRN)-complex was *de novo* expressed. MRE11 is an indirect interaction partner of MSH3 which together play a role in the removal of Holliday junctions [54]. The RAD50:Mre11 complex is also required for the repair of hairpin-capped double-strand breaks and prevents chromosome rearrangements [55]. MRE11 is commonly inactivated in MMR-deficient cancers [56], [57]. Overexpression of MRE11 is a fingerprint of DNA damage response and observed upon radio- and chemotherapy [57]. RAD50 but not NBS1 (nibrin) were induced in the nucleus, which is in line with elevated RAD50 and MRE11 levels. These findings were underlined by an increase in DSBs as revealed by the comet assay. There may be a link of MSH3 and the repair of DSBs because of the PCNA-binding domain of MSH3 [58] and Msh3 recognizes also branched DNA structures with a free 3′ tail in S. cerevisiae [59]. DSBs may lead to oncogenic transformation of mammalian cells [60]. However, silencing of MSH3 did not result in anchorage-independent growth analyzed by soft agar assay.

MSH3-silencing also altered the expression levels of 7 components of the apoptotic pathway among which plectin-1 (a cross-linking protein which organizes the cytoskeleton maintaining its physical stability) and cytoplasmic dynein light chains 1 and 2 (DLC1 and DLC2) showed induced expression levels. The role of plectin-1 and DLCs in cancer development, however, is controversial [61]–[63]. Key factors controlling apoptosis are regulated by the 26S proteasome complex. The 26S protease regulatory subunit 7, which is involved in the ATP-dependent degradation of ubiquitinated protein, is upregulated upon MSH3-deficiency, an effect which may participate in the induction of apoptosis. Vimentin, a type III intermediate filament protein, which is commonly methylated in CRC was induced upon MSH3-silencing [64] and BAX, one of the proapoptotic members of the Bcl-2 family, was upregulated. Mutations in BAX have been shown to mediate tumor progression in later stages of CRC with MSI [65].

Several proteins involved in cell metabolism were repressed upon MSH3-silencing. Asparagine synthetase (an enzyme that generates asparagine from aspartate), biliverdin reductase A (a regulator of glucose metabolism), NADH dehydrogenase (an important enzyme of the mitochondrial electron transport chain), nicotinamide mononucleotide adenylyltransferase 1 (a member of the nicotinamide-nucleotide adenylyltransferases, which are involved in important metabolic redox reactions, protein ADP-ribosylation, histone deacetylation, and in some Ca(2+) signaling pathways) and cytochrome C oxidase subunit 2 (a component of the respiratory chain which is involved in the transfer of electrons from cytochrome c to oxygen) were significantly downregulated suggesting impairments in cell metabolism. However, as measured by flow cytometry cell count itself was not affected. In parallel to the activation of proapoptotic and the repression of metabolic proteins, a number of proteins involved in tRNA aminoacylation and gene expression were also impaired by MSH3-silencing. Leucyl-tRNA synthetase, histidyl-tRNA synthetase, asparaginyl-tRNA synthetase, and 40S ribosomal protein S15 were repressed indicating impairment of protein biosynthesis. However, the changes we observed within the proteome may be correlated with the lack of oncogenic transformation of HCECs by MSH3-silencing.

To maintain growth in culture, primary human colonic epithelial cells (HCEC) were transduced with the catalytic component of human telomerase (hTERT) and cyclin-dependent kinase 4 (Cdk4) [41]. Indeed, a certain amount of HCECs (∼0.1%) lead to the formation of colonies independent of MSH3-silencing. Thus, they cannot be considered as completely normal cells and our proteome findings may be biased by this methodology. Our data are limited within the [AAAG]17- and the [CA]13-repeat (as control); furthermore, it remains unknown if mononucleotide repeats are influenced by MSH3-silencing utilizing our system as another report claims that such repeats are affected by loss or reintroduction of MSH3 [66], [67]. Interestingly, overexpression of the MSH3 gene severely affects the ratio of MutSα to MutSβ and as a result leads to an impaired repair of base/base mismatches followed by a strong mutator phenotype [36]. Furthermore, MLH1 and MSH2 deficiencies strongly correlate with elevated MSI within mononucleotide repeats and therefore loss of such MMR proteins may participate in the loss of tumor suppressor genes which include exonic mononucleotide repeats (such as TGFBR2). Another shortcoming of the proteomic approach is the actual detection threshold, which limits the detection of low abundance proteins. In fact, neither MSH3 nor its binding partner MSH2 was detected by shot-gun proteomics. EMAST is also associated with immune cell infiltration and suggests that inflammation may play a role for its development [16] and increased amounts of CD8+ T lymphocytes were found in tumor cell nests and the tumor stroma in both MSI and EMAST tumors [29]. It would be interesting to investigate the impact of CD8+ T lymphocytes on the stability of EMAST-loci using an *in vitro* co-culture system.

In summary, our study confirms that MSH3-deficiency in human colon epithelial cells results in elevated instability within tetranucleotide repeats and to some extent also in dinucleotide repeats. MSH3-deficiency promotes significant changes within the proteome, which are insufficient to induce oncogenic transformation but rather elicit a DNA-damage response. These data are in parallel with recent observations that loss of MSH3 is associated with DSBs [37] and a lower rate of nodal involvement with a better postsurgical outcome [30]. Further studies including the effect of MSH3-silencing on other repeats as well as a possible enhancer-effect under MLH1- or MSH2-deficient conditions are needed for a better understanding of the consequences of MSH3-deficiency in certain types of CRC.

## Materials and Methods

### Cell Culture

HCT116 (ATCC no. CCL-247), HCT116+chr3 [68] and RKO (ATCC no. CRL-2577) cells were cultured in IMDM (Invitrogen, Vienna, Austria) containing 10% fetal bovine serum (Biochrom, Berlin, Germany). The medium for HCT116+chr3 cells was supplemented with 400 µg/ml geneticin (G418, GIBCO-Invitrogen, Vienna, Austria). All cell lines were maintained at 37°C, 95% humidity, and 5% CO_2_. HCEC were cultured in basal × media (DMEM: M199,4∶1; GIBCO, Eggenstein, Germany), supplemented with EGF (20 ng/mL; BD Biosciences, Heidelberg, Germany), hydrocortisone (1 µg/mL; Sigma, Deisenhofen, Germany), insulin (10 µg/mL), transferrin (2 µg/mL), sodium selenite (5 nM) (all from Gibco, Life Technologies GmbH, Karlsruhe, Germany), 2% cosmic calf serum (HyClone, Bonn, Germany), and gentamicin sulfate (50 µg/mL; Sigma, Deisenhofen, Germany). Cells were cultured in Primaria flasks (Becton Dickinson, Heidelberg, Germany). For selection of stable cell clones transfected with the tetranucleotide frameshift-reporter plasmid pIREShyg2-EGFP-[AAAG]17 cells were cultured in basis media containing additionally 300 µg/ml hygromycin B (Invitrogen, Karlsruhe, Germany). For selection of stable integration of shRNA-vectors (HuSH technology, Origene) cells were cultured in basis media containing additionally 15 µg/ml puromycin (Sigma, Deisenhofen, Germany).

### Chemicals and Media

Inorganic salts, dimethyl sulfoxide (DMSO), ethidium bromide, NaOH, Trizma base, Triton X-100, trypan blue, H_2_O_2_, ethylenediaminetetraacetic acid disodium salt dehydrate (Na_2_EDTA) were purchased from Sigma-Aldrich (Steinheim, Germany). Dulbeccós PBS came from PAA Laboratories GmbH (Pasching, Austria). Low melting point agarose and normal melting point agarose (LMA and NMA) were obtained from Gibco (Paisley, UK). Crystal violet was purchased from Merck (Germany).

### Generation of the pIREShyg2-EGFP-[AAAG]17 Frameshift Reporter Plasmid

The plasmid pIREShyg2-EGFP allows the expression of EGFP under the control of a constitutive CMV promoter [42]. For generation of pIREShyg2-EGFP-[AAAG]17, which shifts the EGFP reading frame into a −1 bp position, pIREShyg2-EGFP was linearized with PmeI (generating a 3′ blunt end) and AscI (generating a 5′ GCGC-overhang). Compatible DNA repeat oligonucleotides of [AAAG]17 were generated by hybridization of forward and reverse single DNA oligonucleotides with a 5′ GCGC-overhang and a 3′ blunt end. After ligation, the product was transformed into Stbl2 competent bacteria (Gibco, Life Technologies GmbH, Karlsruhe, Germany). Amplified plasmids were isolated and sequenced using EGFP-specific primers flanking the DNA repeat sequence.

### Establishment and Characterization of Frameshift Reporter Cell Lines

MLH1-proficient and MLH1-deficient cells (HCT116+chr3, HCT116) and primary colon epithelial cells (HCEC-1CT) [41] were transfected with pIREShyg2-EGFP-[AAAG]17 similar to our previous experiments using pIREShyg2-EGFP-[CA]13 [42]. Single cell clones were selected and characterized by sequencing (only for HCT116 and HCT116+chr3), Southern blotting and flow cytometry (Figure 1). HCEC-1CT transfected with pIREShyg2-EGFP-[AAAG]17 were used as mixed populations since primary cells need a certain cell density and cell to cell contact for proper growth precluding single cell cloning. Cells were selected with 300 µg/ml hygromycin B and 15 µg/ml puromycin. In addition, DLD-1 and HCT116 cells were transfected with pIREShyg2-EGFP-[CA]13, pIREShyg2-EGFP-[AAAG]17 or the non-repeat vector pIREShyg2-EGFP-[N]26 [40] and selected with 250 µg/ml hygromycin B for three weeks.

### MSH3-silencing in HCEC Frameshift Reporter Cells

HCEC-1CT, HCEC-1CT-[AAAG]17 and HCEC-1CT-[CA]13 cells were transfected with the pRFP-C-RS vectors (Origene) either without shRNA cassette insert (control), a non-effective 29-mer scrambled (scrambled) or a gene-specific shRNA cassette for suppression of MSH3 (shMSH3). RFP-positive (pRFP-C-RS harboring) cells were selected with 15 µg/ml puromycin as well as 300 µg/ml hygromycin B (for frameshift reporter vectors pIREShyg2-EGFP-[CA]13 or pIREShyg2-EGFP-[AAAG]17). Settings for the analysis of mutant fractions were established using non-transfected (non-fluorescent) HCEC-1CT, EGFP-positive HCEC-1CT-[AAAG]17, RFP-positive HCEC-1CT-shMSH3 and RFP/EGFP-positive HCEC-1CT-[AAAG]17-shMSH3 cells. Stable cell lines were analyzed by flow cytometry. >98% of total cells were RFP-positive (data not shown).

### Analysis of Frameshift Mutations by Flow Cytometry

EGFP-negative (M0) frameshift reporter cells were sorted by FACSAria using CloneCyt Plus sorting technology (Becton Dickinson Immunocytometry Systems) into 24-well plates. After 3, 6 or 9 days cells were rinsed with cold Ca^2+^/Mg^2+^-free PBS (GIBCO-Invitrogen, Vienna, Austria) and detached with 160 µl Accutase (PAA Laboratories, Linz, Austria). 120 µl of the cell suspension were directly analyzed on a FACScan and analyzed using CellQuest (Beckton Dickinson). Cell counts were multiplied by 2.0 to quantify the total number of cells per well. Populations of HCT116 and HCT116+chr3 derivatives displaying no EGFP-fluorescence were named M0 (no mutations), populations with low fluorescence intensity M1 (intermediate mutations), and those with high fluorescence intensity M2 (definitive mutations). The counts of M1 and M2 cells were expressed as percentage of R1 (total cell number). In HCEC-1CT derivatives a discrimination of M1 and M2 was not possible due to double fluorescence (red/green). For HCEC 1CT-derived reporter cell lines only the total EGFP-positive mutant fraction was analyzed.

### Sequence Analysis

Single cell clones of M0, M1, and M2 populations of HCT116 and HCT116+chr3 cells containing the [AAAG]17-reporter plasmid were sorted into 96-well plates using FACSAria and cultured for several days to obtain approximately 50–100 cells per clone. The medium was removed and cells were immediately lysed with 50 mM NaOH and boiled for 10 min at 99°C. The microsatellite locus of the pIRES-hyg2-EGFP-[AAAG]17 vector was amplified by PCR and further subjected to cycle sequencing as described above to detect the type of frameshift mutations which occurred in the respective microsatellite.

### Western Blot Analysis

Cell lysates were obtained as described [69]. For western blotting, 50 µg of lysates were loaded on gradient polyacrylamide gels and membranes were incubated with antibodies against MSH3 (sc-11441, rabbit polyclonal, Santa Cruz, CA, USA), MSH6 (610919, mouse monoclonal, BD Biosciences, San Jose, CA) and α-tubulin (ab7291, mouse monoclonal, Abcam, Cambridge, MA). As secondary antibodies we used IRDye 680 (anti-mouse) and IRDye 800 (anti-rabbit) conjugated secondary antibodies (LI-COR Biosciences, Bad Homburg, Germany). Signals were detected and quantified using the Odyssey infrared imaging system (LI-COR Biosciences).

### Statistical Analyses of Mutation Rates

The mutation rate of the [AAAG]17 repeat in HCT116 and HCT116+chr3 cells was calculated as described before [40]. Data from M2 cells from the last day of analysis were used to calculate the mutation rate by the method of the mean and the maximum likelihood approaches. A cloning efficiency of 20% was considered for the estimation of the mutation rates. In order to assess the difference between cell lines, the mutation rates of clone replicates were compared by Welch Two Sample t-test. The Lea-Coulson method of the median was used to calculate the mutation rates of single clones as it is independent of the number of clone replicates. The resulting p-values were adjusted for multiple testing using the Benjamini-Hochberg method to control the false discovery rate. Experiments were carried out in triplicates and repeated twice. Data are represented as mean with the SD and compared by using the Student's t test or one-way ANOVA. P-values of <0.05 were considered to be statistically significant.

### Cell Fractionation

The isolation of cytoplasmic proteins was performed as described by Gundacker et al. [33]. Cells were lysed in hypotonic lysis buffer (10 mM HEPES/NaOH, pH 7.4, 0.25 M sucrose, 10 mM NaCl, 3 mM MgCl_2_, 0.5% Triton X-100) supplemented with protease inhibitors and pressed through a 23 g syringe to induce cell lysis. The cytoplasmic fraction was separated from nuclei by centrifugation and precipitated by the addition of ethanol. The remaining pellet was lysed with 100 mM Tris/HCl, pH 7.4, 1 mM EDTA, pH 7.5, 500 mM NaCl for 10 min on ice and afterwards resuspended with 10 mM Tris/HCl, pH 7.4, 1 mM EDTA, pH 7.5, 0.5% NP-40 and kept on ice for 15 min to obtain the nuclear extract. After centrifugation the protein was precipitated again by the addition of ethanol. Afterwards, all protein samples were dissolved in sample buffer (7.5 M urea, 1.5 M thiourea, 4% CHAPS, 0.05% SDS, 100 mM DDT).

### 1-D PAGE for Subsequent Shotgun Analysis

Protein fractions were loaded on 13% polyacrylamide gels; electrophoresis was performed until complete separation of a pre-stained molecular marker (Dual Color, Biorad) was visible. After fixation with 50% methanol/10% acetic acid and subsequent silver staining, gel lanes were cut out of the gel and digested with trypsin as described below.

### Tryptic Digest

Protein spots, 1-D bands, were cut out of the gel, the gel pieces were de-stained with 15 mM K_3_Fe(CN)_6_/50 mM Na_2_S_2_O_3_ and extensively washed with 50% methanol/10% acetic acid. The pH was adjusted with 50 mM NH_4_HCO_3_, proteins were reduced with 10 mM DTT/50 mM NH_4_HCO_3_ for 30 min at 56°C and alkylated with 50 mM iodacetamide/50 mM NH_4_HCO_3_ for 20 min in the dark. Afterwards the gel-pieces were treated with ACN and dried in a vacuum centrifuge. Between each step, the tubes were shaken 5–10 min (Eppendorf Thermomixer comfort). Dry gel spots were treated with trypsin, 0.1 mg/mL (Trypsin, sequencing grade, Roche Diagnostics, Germany)/50 mM NH_4_HCO_3_, in a ratio of 1∶8 for 20 min on ice, afterwards covered with 50 mM NH_4_HCO_3_ and were subsequently incubated overnight at 37°C. The digested peptides were eluted by adding 50 mM NH_4_HCO_3_, the supernatant was transferred into silicon-coated tubes and this procedure was repeated two times with 5% formic acid/50% ACN. Between each elution step the gel-spots were ultrasonicated for 10 min. Finally the peptide solution was concentrated in a vacuum centrifuge to an appropriate volume.

### MS Analysis

MS was performed as described previously [24]. Peptides were separated by nano-flow LC (1100 Series LC system, Agilent, Palo Alto, CA, USA) using the HPLC-Chip technology (Agilent) equipped with a 40 nL Zorbax 300SBC18 trapping column and a 75 mm × 150 mm Zorbax 300SBC18 separation column at a flow rate of 400 nL/min, using a gradient from 0.2% formic acid and 3% ACN to 0.2% formic acid and 50% ACN over 60–80 min. Peptide identification was accomplished by MS/MS fragmentation analysis with an iontrap mass spectrometer (XCT-Ultra, Agilent) equipped with an orthogonal nanospray ion source. The MS/MS data, including peak list generation and search engine, were interpreted by the Spectrum Mill MS Proteomics Workbench software (Version A.03.03, Agilent) allowing for two missed cleavages and searches against the SwissProt Database for human proteins (Version 12/2010 containing 20328 protein entries) allowing for precursor mass deviation of 1.5 Da, a product mass tolerance of 0.7 Da and a minimum matched peak intensity (%Scored Peak Intensity) of 70%. Due to previous chemical modification, carbamidomethylation of cysteine was set as fixed modification.

Oxidation of methionine was the only variable modifications considered here. All data including peptide sequences, peptide scores, MS2 spectra, sequence coverage, second hits and search results using the reversed database are fully documented in the corresponding PRIDE XML files. For peptides scoring above 13.0, consistently less than 1% matched using the reversed database compared to the true database. Peptides scoring between 9 and 13 were only included if they matched to a corresponding peptide scoring >13 in our database. The false discovery rate is therefore less than 1%. Data interpretation was done using the Griss Proteomics Database Engine (GPDE) [70]. The PRIDE accession numbers for the proteomics data are 22152–22160. The database can be accessed at http://www.ebi.ac.uk/pride.

### Soft Agar Colony Formation

HCT116, RKO, HCEC-1CT, HCEC-1CT-[AAAG]17-control, HCEC-1CT-[AAAG]17-scrambled and HCEC-1CT-[AAAG]17-shMSH3 were cultured in six-well plates with a 0.35% top agar layer. The base- (0.5%) and top agar for HCT116 and RKO cells were prepared with 2X IMDM while for HCEC-1CT cells and its derivatives the medium was changed to HCEC-specific medium containing appropriate components as described above. 5×10^4^ cells were seeded within the top layer of the soft agar. The plates were incubated for 14 days and colonies were counted.

### Single Cell Gel Electrophoresis Assay (SCGE Assay/comet Assay)

The experiments were carried out according to the international guidelines for comet assays [71], [72]. Cells (5×10^5^ per tube) were cultured in PBS (pH 7.4) in Eppendorf tubes (Eppendorf AG, Hamburg, Germany). Additionally, the cells were mixed with 0.5% LMA and transferred to agarose coated slides (1.0% NMA). Slides were immersed in lysis solution (pH 10, 0.1 M Na_2_EDTA, 2.5 M sodium chloride, 0.10 M Trizma base, prior to use 1% Triton X-100 and 10% dimethyl sulfoxide were added freshly) overnight at 4°C. Subsequently, the slides were placed in a horizontal gel electrophoresis tank and DNA was allowed to unwind for 20 min in electrophoresis buffer (0.3 M NaOH and 1 mM Na_2_EDTA, pH>13). Additionally electrophoresis was carried out for 20 min (300 mA, 1.0 V/cm corresponding to 25 V) at 4°C. Neutralization buffer (0.4 M Trizma base, pH 7.5) was used to wash (two times for 8 min) and neutralize the electrophoresis buffer. Slides were rinsed in distilled water and air-dried overnight. The DNA was stained with ethidium bromide (20 µg/mL) and the percentage of DNA in the tail was analyzed with a computer aided system (Comet Assay IV, Perceptive Instruments, UK).

### Statistical Analysis

Values are expressed as mean±SD of one experiment as a graphical representation. Three independent experiments were conducted. Statistical significance was tested by non-parametric unpaired t-Test, p-values≤0.05 were considered as significant. Statistical analyses were performed using Graphpad Prism 4.0 (Graphpad Software, San Diego, CA).

## Supporting Information

Figure S1 **MSH6-deficiency induces low levels of MSI within [AAAG]17 repeats.** HCT116 and DLD-1 cells were transfected with pIREShyg2-EGFP-[AAAG]17, pIREShyg2-EGFP-[CA]13, and the control non-repeat plasmid pIREShyg2-EGFP-[N]26. Stable cells were selected and EGFP-negative populations were sorted into 24-well plates. After 7 days the mutant fraction was analyzed by flow cytometry. Data represent mean±SD from three experiments. Stars indicate statistical significance (P<0.05).
(TIF)

Table S1 Protein expression levels upon MSH3-silencing analyzed by shotgun proteomics. For analysis we pooled HCEC-1CT and HCEC-1CT-[AAAG]17-scrambled (Pool A, reference, columns K to Q) as well as HCEC-1CT-[AAAG]17-shMSH3 and HCEC-1CT-shMSH3 (Pool B, analysis, columns D to J). Column R shows induced and column S repressed proteins. n = relative nuclear expression, c = relative cytoplasmatic expression, N = nuclear induction, C = cytoplasmatic induction, nc = nuclear and cytoplasmatic relative expression, NC = nuclear and cytoplasmatic induction. In columns A to C proteins which are either induced/completely repressed or proteins which revealed increased or decreased expression levels of at least 3-fold are marked with an asterisk in columns R and S.
(XLSX)

Table S2 Over-represented pathways of over-expressed/induced proteins upon MSH3-silencing. Reactome skypainter was used to analyze protein levels changed by MSH3-silencing and their relation to certain cellular pathways. Pathways which map with a p-values below 1×10^−4^ where considered as highly significant (marked with an asterisk in column E).
(XLSX)

Table S3 Over-represented pathways of repressed proteins upon MSH3-silencing. Reactome skypainter was used to analyze protein levels changed by MSH3-silencing and their relation to certain cellular pathways. Pathways which map with a p-values below 1×10^−4^ where considered as highly significant (marked with an asterisk in column E).
(XLSX)
