# Supplementary figures and images for: MSH3-Deficiency Initiates EMAST without Oncogenic Transformation of Human Colon Epithelial Cells
Source: PLoS One. 2012 Nov 27;7(11):e50541. doi: 10.1371/journal.pone.0050541 (PMC3507781; doi:10.1371/journal.pone.0050541)

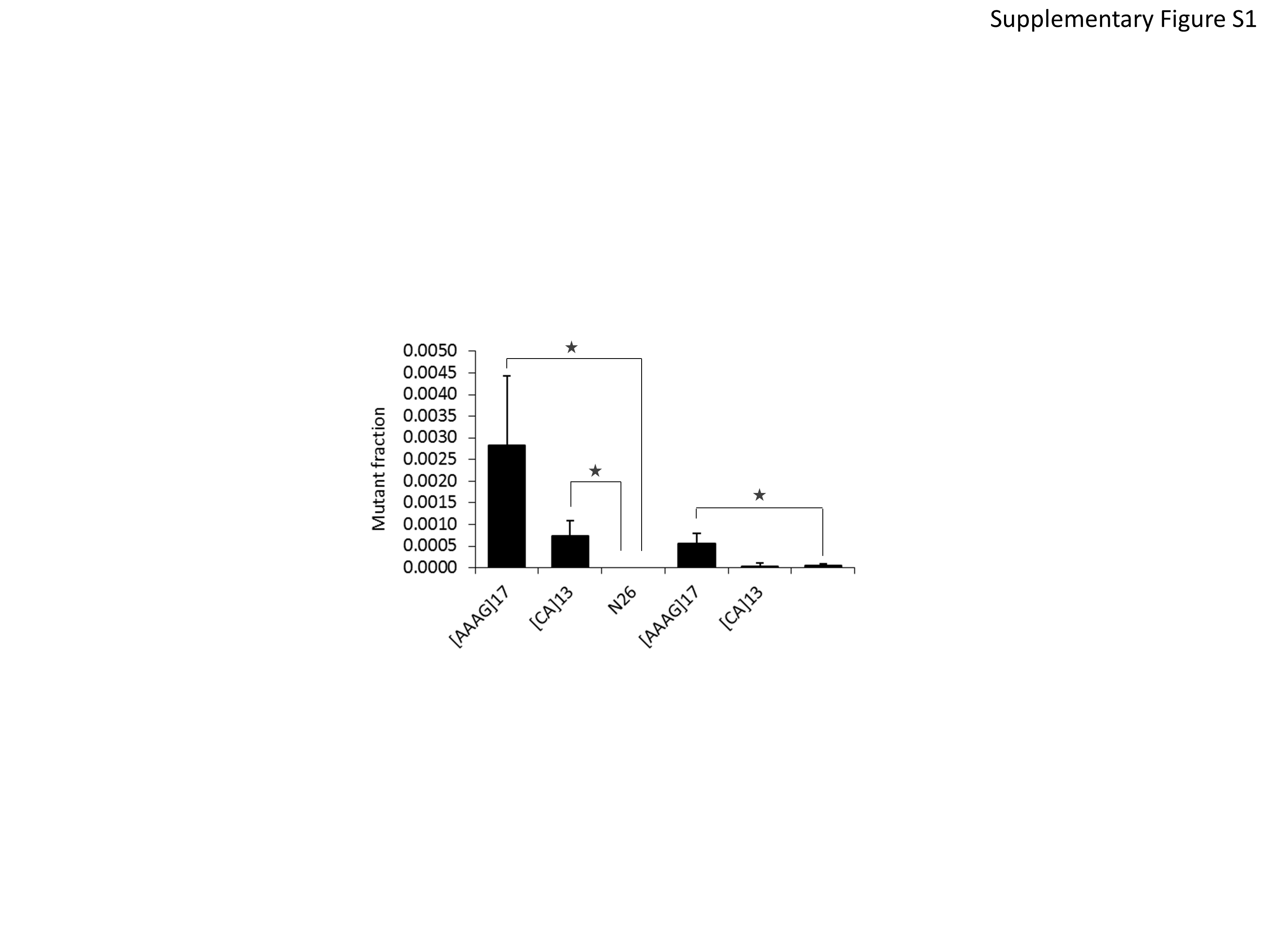

Supplement: Figure S1 — MSH6-deficiency induces low levels of MSI within [AAAG]17 repeats. HCT116 and DLD-1 cells were transfected with pIREShyg2-EGFP-[AAAG]17, pIREShyg2-EGFP-[CA]13, and the control non-repeat plasmid pIREShyg2-EGFP-[N]26. Stable cells were selected and EGFP-negative populations were sorted into 24-well plates. After 7 days the mutant fraction was analyzed by flow cytometry. Data represent mean±SD from three experiments. Stars indicate statistical significance (P<0.05). (TIF) [file pone.0050541.s001.tif]
